# YOLOv11-WBD: A wavelet-bidirectional network with dilated perception for robust metal surface defect detection

**DOI:** 10.1371/journal.pone.0331025

**Published:** 2025-09-10

**Authors:** Li Guan, Haitao Zhang, Yijun Zhou, Xinyu Du, Mingxuan Li

**Affiliations:** 1 Department of Smart Manufacturing, Industrial Perception and Intelligent Manufacturing Equipment Engineering Research Center of Jiangsu Province, Nanjing Vocational University of Industry Technology, Nanjing, Jiangsu, China; 2 Department of Data Analysis, Nanjing Weiwo Software Technology Co., Ltd. Nanjing, Jiangsu, China; Chongqing Normal University, CHINA

## Abstract

In the field of quality control, metal surface defect detection is an important yet challenging task. Although YOLO models perform well in most object detection scenarios, metal surface images under operational conditions often exhibit coexisting high-frequency noise components and spectral aliasing background textures, and defect targets typically exhibit characteristics such as small scale, weak contrast, and multi-class coexistence, posing challenges for automatic defect detection systems. To address this, we introduce concepts including wavelet decomposition, cross-attention, and U-shaped dilated convolution into the YOLO framework, proposing the YOLOv11-WBD model to enhance feature representation capability and semantic mining effectiveness. To improve robustness, a plug-and-play Wavelet-Attentive Multiband Fusion Module (WAMF) is designed, achieving decoupling of low-frequency and high-frequency features and adaptive multi-frequency fusion. To effectively aggregate multi-scale features, a Bottleneck-Enhanced Dilated U-Conv Module (BEDU) is designed, fusing global and local information with lower computational resource consumption. To address feature fusion, a Bidirectional Depthwise Cross-Attention Module (BDCA) is designed to replace simple concatenation and convolution operations, achieving adaptive feature fusion. YOLOv11-WBD undergoes rigorous evaluation on the public NEU-DET and GC10-DET datasets; experimental results show that the improved model achieves performance gains on both datasets: the mAP@0.5 metric increased by 5.8% and 2.8% respectively. Furthermore, the improved model demonstrates stronger noise tolerance, maintaining high defect detection capability even in moderate noise environments, providing a valuable solution for industrial applications.

## 1. Introduction

With the rapid development of modern industry, metal has become an indispensable core material for most industries. However, the complexity of manufacturing processes may lead to various defects in products, such as cracks, holes, corrosion and other types of defects [1,2]. These defects not only cause quality losses but may also trigger chain failures. Traditional metal surface defect detection relies on manual inspection, a method that is labor-intensive and time-consuming. Moreover, due to differences in personnel skill and experience, it can easily lead to inconsistent results such as false positives and missed detections [3]. Therefore, developing robust and high-precision metal surface defect detection models deployable on edge devices is of decisive significance for ensuring production quality and improving operational efficiency.

Compared with traditional manual inspection, the development of computer vision technology provides a more efficient solution for this field. Among them, deep learning-based object detection algorithms have attracted considerable attention due to their outstanding performance in image processing and computer vision tasks [4–6]. Algorithms driven by deep learning can autonomously learn and extract complex feature representations, making them particularly suitable for metal surface defect recognition tasks [7]. Such models can be integrated into real-time production lines to enhance production efficiency and ensure product quality stability [8,9].

**Fig 1:**
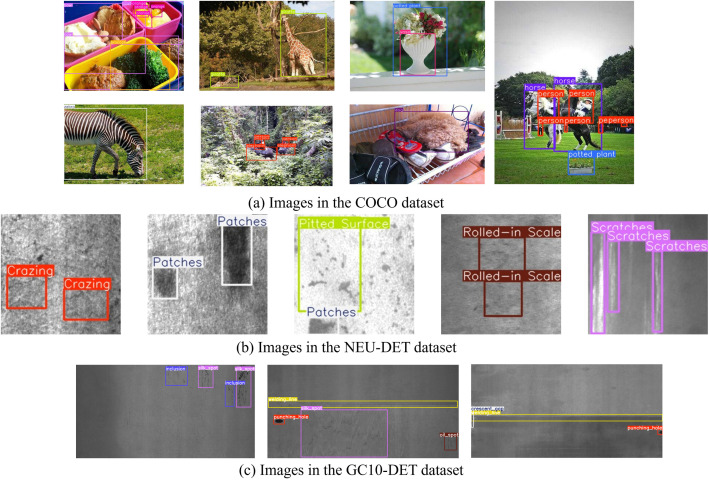
Comparative visualization of defect detection versus conventional object detection.

However, different from object detection in general images, metal surface images, as shown in Fig 1, contain a large number of small-scale, weakly contrasted, multi-class defect targets whose spatial distribution typically exhibits sparsity. Furthermore, affected by insufficient lighting and dust interference commonly present in operational conditions, metal surface images often exhibit coexisting high-frequency noise components and spectral aliasing background textures [10]. These characteristics not only increase the complexity of feature extraction but may also lead to the loss of fine-grained details and critical features, ultimately limiting the model’s detection performance and reducing detection accuracy.

Related research indicates that preprocessing images under operational conditions and increasing model size can improve detection accuracy [11,12]. However, such technical approaches significantly increase computational burden, posing challenges for deploying models on edge devices. To overcome the above limitations, we designed a metal surface defect detection model named YOLOv11-WBD, aiming to strike a balance between detection accuracy, model complexity, and model robustness. The main contributions are as follows:

1. Wavelet-Attentive Multiband Fusion Module (WAMF): This module enables decoupling of low-frequency and high-frequency features and adaptive multi-band fusion, reducing interference from high-frequency noise and enhancing model robustness.
2. Bottleneck-Enhanced Dilated U-Conv Module (BEDU): This module combines bottleneck convolution, dilated convolution, and U-shaped skip connections to achieve effective fusion of global and local information, enhancing the model’s ability to capture low-contrast and tiny targets.
3. Bidirectional Depthwise Cross-Attention Module (BDCA): This module employs a cross-attention mechanism for information fusion across different feature levels, achieving semantic information complementarity, thereby further enhancing the model’s feature extraction capability.

The subsequent structure of this paper is organized as follows: Chapter 2 outlines current metal surface defect detection methods and analyzes their limitations; Chapter 3 elaborates on the network structure and working principles of YOLOv11-WBD; Chapter 4 evaluates the feasibility and effectiveness of the algorithm in industrial applications through experiments; Chapter 5 concludes the paper.

## 2. Related works

With the rapid development of deep learning technology, convolutional neural networks have been increasingly widely applied in object detection tasks. Represented by two-stage detection algorithms like Faster R-CNN, their high-precision characteristics make them prominent in surface defect detection. For example:

Wang [13] proposed an improved Faster R-CNN-based detection algorithm for identifying typical surface defects in contact lenses, achieving an average precision of 86.95% compared to the original Faster R-CNN. Zheng [14] addressed the challenge of wafer surface defects being easily confused with the background by proposing a novel method combining background differencing with improved Faster R-CNN, increasing average precision by 5.2% compared to the previous version. Liu [15] replaced the original feature extraction network in Faster R-CNN with ResNet50 for recognizing sprouting and other damages on potato surfaces, improving average precision by 7.79% over the original Faster R-CNN.

Although these methods demonstrate excellent defect recognition accuracy, their low detection speeds struggle to meet real-time requirements in industrial scenarios. In contrast, single-stage detection algorithms represented by the YOLO series treat object detection as an end-to-end regression problem, enhancing detection speed by directly predicting anchor positions on feature maps [16]. In surface defect detection, typical studies include:

Gao [17] proposed Dense-YOLO, a fast surface defect detection network that combines DenseNet with YOLOv3 and reconstructs the feature pyramid network. Experiments show this network achieves real-time detection while maintaining high accuracy. Liang [18] proposed the YOLOv5s-P6SE model, which adds a P6 detection layer for detecting extra-large targets to YOLOv5, incorporates the SE attention module and flexible non-maximum suppression, improving average precision by 5.5% compared to the original YOLOv5s. Li [19] applied the YOLOv8 model to locate and classify defects on commutator surfaces, achieving 98% average precision at 27 frames per second.

**Fig 2:**
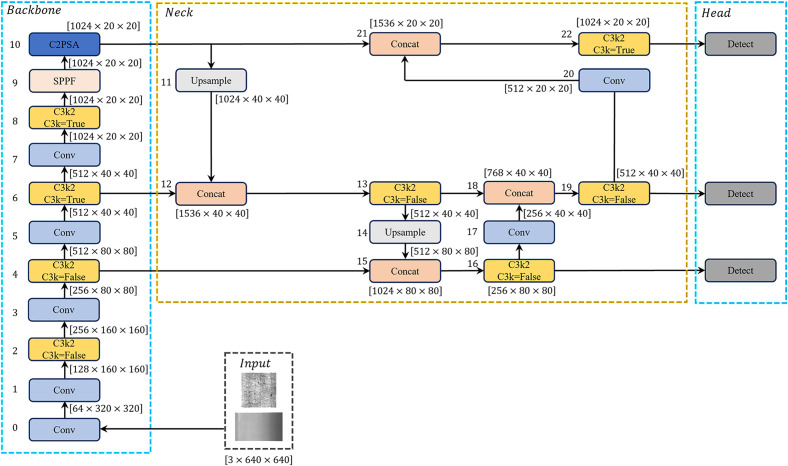
The structure of YOLOv11.

As the latest iteration developed by the Ultralytics team, YOLOv11 [20,21] represents one of the current state-of-the-art object detection algorithms, with its network architecture shown in Fig 2. Compared to previous versions, its main improvements include:

1. Upgrading the original C2f module to the C3k2 module, integrating advantages of both C2f and C3 architectures while optimizing feature extraction paths and gradient propagation mechanisms, significantly reducing parameters and improving computational efficiency;
2. Introducing the C2PSA module after the SPPF module to enhance the model’s adaptability to occluded objects;
3. Employing depthwise separable convolution in the detection head to reduce redundant computations and accelerate model inference efficiency.

Although current single-stage detectors based on the YOLO series demonstrate significant speed advantages, their deployment on edge computing devices still faces challenges including insufficient robustness, high computational complexity, and room for improvement in detection accuracy. To address these issues, this paper proposes a novel object detection network named YOLOv11-WBD for metal surface defect detection. Based on the YOLOv11 architecture, YOLOv11-WBD achieves breakthrough progress in improving detection accuracy and robustness compared to YOLOv11s.

## 3. Methods

### 3.1. YOLOv11-WBD model structure

Although YOLOv11 has been widely applied to various object detection tasks, it still faces the following challenges in the field of metal surface defect detection:

1. Images acquired under operational conditions often exhibit coexisting high-frequency noise components and spectral aliasing background textures, while the existing YOLOv11 lacks sufficient robustness and is susceptible to noise interference.
2. Metal surface defects exhibit complex morphologies, coexist in multiple classes, and have weak contrast with the background, making it difficult for YOLOv11 to capture subtle features and low-contrast targets.

To overcome the above limitations, we propose the YOLOv11-WBD model, which incorporates three core improvement modules.

Firstly, a Wavelet-Attentive Multiband Fusion (WAMF) Module is proposed, coupling traditional image enhancement with the shallow layers of the original YOLOv11 backbone network to achieve decoupling of low-frequency and high-frequency features and adaptive multi-band fusion, thereby reducing interference from high-frequency noise and enhancing model robustness.

Secondly, a Bottleneck-Enhanced Dilated U-Conv (BEDU) Module is designed, employing lightweight bottleneck convolution for feature extraction and combining dilated convolution with U-shaped skip connections to achieve effective fusion of global and local information, thereby enhancing the model’s ability to capture low-contrast and tiny targets.

Additionally, a Bidirectional Depthwise Cross-Attention (BDCA) Module is designed, mapping deep and shallow features to a shared query/key/value embedding space, and then employing a cross-attention mechanism for information fusion across different feature levels to achieve semantic information complementarity, thereby further enhancing the model’s feature extraction capability.

**Fig 3:**
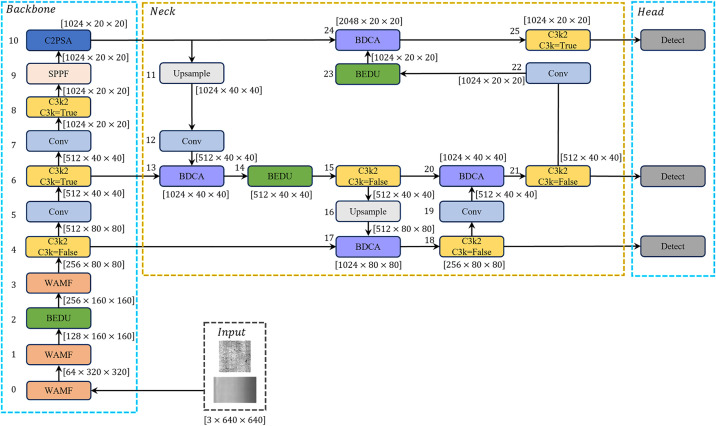
The structure of YOLOv11-WBD.

The network structure diagram of YOLOv11-WBD is shown in Fig 3.

### 3.2. Wavelet-Attentive Multiband Fusion (WAMF) module

Although YOLOv11 demonstrates excellent detection speed and accuracy, the traditional convolution operations in its backbone network lack discriminative capability for high-frequency noise and details, making it susceptible to noise interference, which leads to decreased detection accuracy and insufficient robustness. To address this issue, we propose a Wavelet-Attentive Multiband Fusion (WAMF) Module for preprocessing the input raw image.

The module first extracts low-frequency features and horizontal, vertical, and diagonal high-frequency features through wavelet transform. The computation process is shown in Equation (1):


$$\left\{ \begin{array}{c} \begin{array}{c} LL=\;\left(L_{x}*\left(L_{y}*I\right)\right)\\ \begin{array}{c} LH=\;\left(L_{x}*\left(H_{y}*I\right)\right)\\ HL=\;\left(H_{x}*\left(L_{y}*I\right)\right) \end{array} \end{array}\\ HH=\;\left(H_{x}*\left(H_{y}*I\right)\right) \end{array}\right.\;$$ (1)

where L and H denote the low-pass filter and high-pass filter, respectively; x and y represent the horizontal and vertical directions; and I denotes the input feature map. LL corresponds to low-frequency features (e.g., smooth backgrounds and structural information), LH to horizontal high-frequency features (e.g., edges), HL to vertical high-frequency features, and HH to diagonal high-frequency features (typically dominated by noise).

Subsequently, adaptive multi-band fusion is achieved via a lightweight multi-head attention mechanism. This preserves structural information (e.g., feature morphology) in low-frequency components while separating noise from directional fine details in high-frequency components. Valid high-frequency information is retained through a gating mechanism, as formalized in Equations (2) and (3):


$$W=concat\left(w_{LL},w_{LH},w_{HL},w_{HH}\right)=Softmax\left(\frac{QK^{T}}{\sqrt{d}}\right)V$$ (2)

$$fused=w_{LL}\times LL+w_{LH}\times LH+w_{HL}\times HL+w_{HH}\times HH$$ (3)

Here, Q,K and V denote the sub-band feature mappings, while fused represents the integrated feature after dynamically suppressing noise and enhancing defects via the gating mechanism.

**Fig 4:**
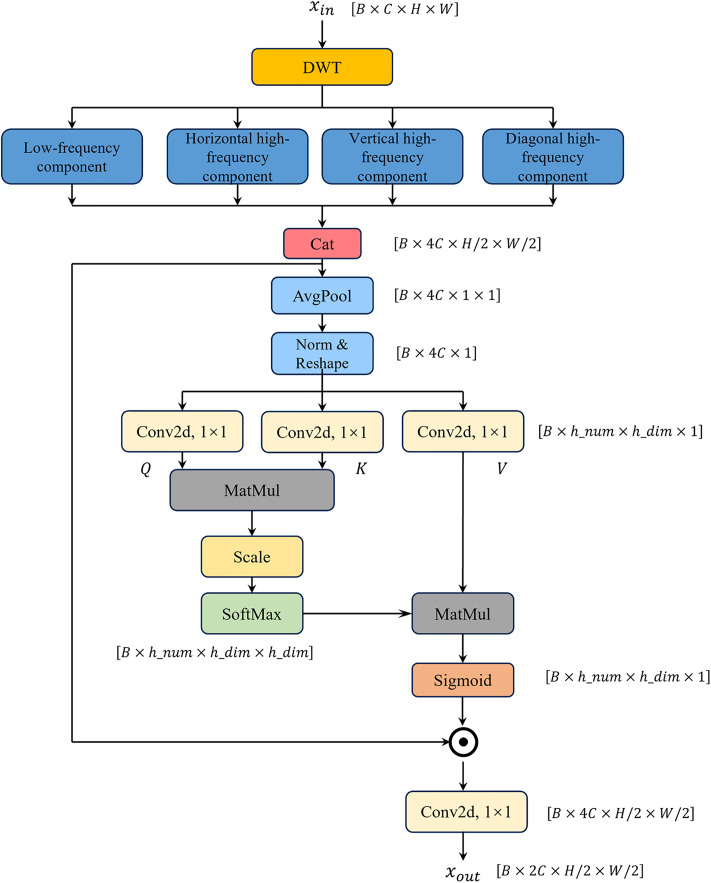
Wavelet-Attentive Multiband Fusion (WAMF) module.

The WAMF module is applied to the shallow layers of the backbone network in YOLOv11-WBD, designed to preprocess the original image. The wavelet transforms employed by this module intrinsically achieves feature down sampling and increases channels, partially replacing the original YOLOv11 backbone network’s approach of adjusting feature map dimensions through convolution stride and kernel number settings. The detailed network architecture is shown in Fig 4.

The WAMF module integrates traditional wavelet transform and multi-head attention mechanisms. After down sampling in the wavelet transform algorithm, the width and height dimensions of the original feature map are halved. After concatenating the decomposed features, the feature map becomes [B,4C,H/2,W/2]. Subsequently, average pooling layers and multi-head attention mechanisms are connected in series to achieve adaptive fusion of multi-frequency features along the channel dimension.

### 3.3. Bottleneck-Enhanced Dilated U-Conv (BEDU) module

Target shapes and textures in industrial inspection images are often complex and exhibit weak contrast with the surrounding background. Balancing computational resources and recognition accuracy is difficult through the traditional approach of stacking ordinary convolution layers. To address this issue, we propose a Bottleneck-Enhanced Dilated U-Conv (BEDU) Module to effectively fuse global and local information.

The module first captures scene information over large ranges through dilated convolution with a large receptive field (e.g., dilation = 2), establishing long-range dependencies between defects and the background. The receptive field calculation for dilated convolution is given by Equation (4):


R=(K−1)×d+1 (4)

where R denotes the receptive field size corresponding to a point on the output feature map, K represents the original kernel size of the dilated convolution, and d indicates the dilation rate controlling the spacing between kernel elements.

Subsequently, lightweight bottleneck structures enhance local details to address the issue of blurred fine-grained defects (e.g., micro-crack edges) when using dilated convolution alone. The computation process is formalized in Equations (5) and (6):


$${\text{F}}_{\text{comp}}={\text{Conv}}_{\text{1}\times \text{1}}\left({\text{F}}_{\text{in}},{\text{C}}_{\text{in}}\to \frac{{\text{C}}_{\text{in}}}{\text{r}}\right)$$ (5)

$${\text{F}}_{\text{out}}={\text{Conv}}_{\text{1}\times \text{1}}\left(\text{DepthwiseConv}\left({\text{F}}_{\text{comp}}\right),\frac{{\text{C}}_{\text{in}}}{\text{r}}\to {\text{C}}_{\text{in}}\right)$$ (6)

Here, r denotes the compression ratio, ${Conv}_{\text{1}\times \text{1}}(\cdot )$ represents pointwise convolution, $F_{in}$ and $C_{in}$ indicate the input feature map and its channel count, respectively, and DepthwiseConv(·) denotes depthwise convolution.

Finally, contextual information is fully utilized through a U-shaped structure and skip connections, enabling effective fusion of global and local information.

**Fig 5:**
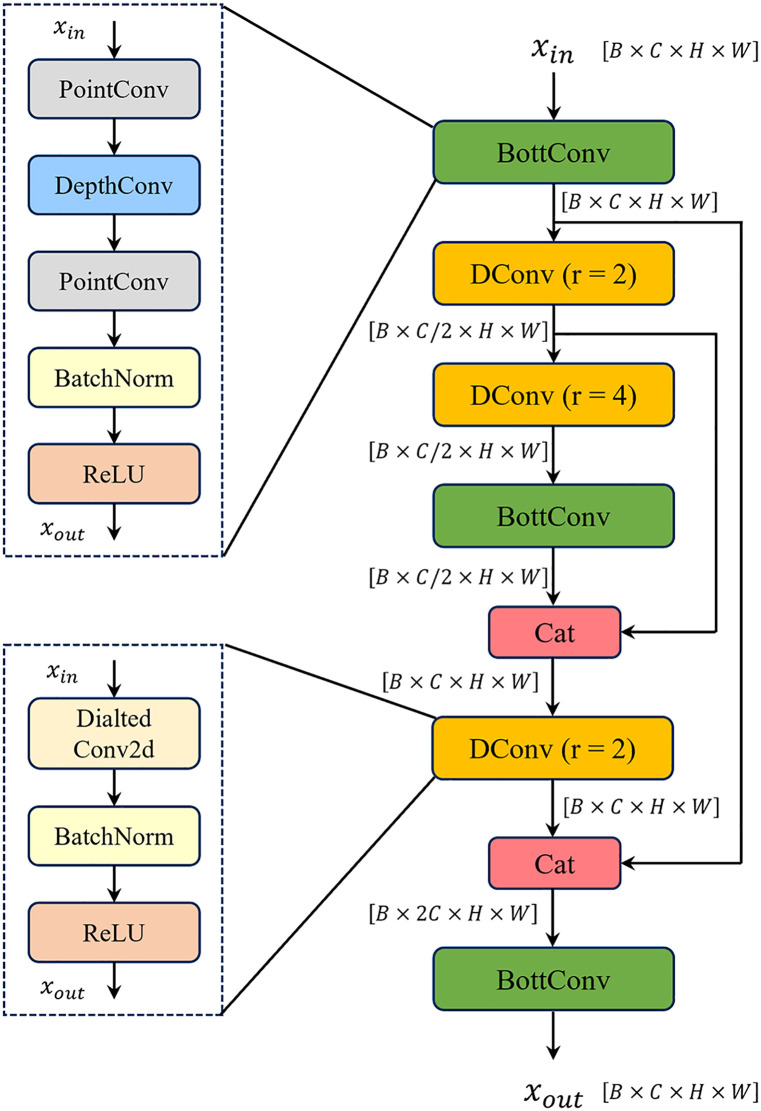
Bottleneck-Enhanced Dilated U-Conv (BEDU) module.

The BEDU module is used in the fusion part of the neck network and the shallow layers of the backbone network in YOLOv11-WBD. The Bottle-Conv within this module employs pointwise convolution and depth-wise convolution to replace traditional convolution, reducing computational load and parameters. Furthermore, the concatenated dilated convolutions coupled with the skip connections of the U-shaped structure can alleviate the information loss problem caused by dilated convolution. The detailed network architecture is shown in Fig 5.

The BEDU module does not alter the dimensions of the original feature map; therefore, it can also serve as a plug-and-play module for other vision tasks.

### 3.4. Bidirectional Depthwise Cross-Attention (BDCA) module

Industrial inspection requires full utilization of image contextual information to accurately determine target categories and locations. However, in YOLOv11, traditional concatenation operations struggle to effectively fuse shallow and deep features. To address this issue, we propose a Bidirectional Depthwise Cross-Attention (BDCA) Module, enabling complementary integration of local shallow features and semantic deep features.

This module maps deep and shallow features to a shared query/key/value embedding space using depthwise separable convolution, then performs cross-attention computations in both “deep → shallow” and “shallow → deep” directions, with the concatenated results serving as output. This allows the module’s output to retain complementary semantic information from heterogeneous feature levels while maintaining computational efficiency. The computational process of this structure is formalized in Equations (7), (8), and(9):


$${\text{fused}}_{\text{shallow}\to \text{deep}}=\text{Softmax}\left(\frac{{\text{Q}}_{\text{shallow}}{\text{K}}_{\text{deep}}^{\text{T}}}{\sqrt{\text{d}}}\right)\cdot {\text{V}}_{\text{deep}}$$ (7)

$${\text{fused}}_{\text{deep}\to \text{shallow}}=\text{Softmax}\left(\frac{{\text{Q}}_{\text{deep}}{\text{K}}_{\text{shallow}}^{\text{T}}}{\sqrt{\text{d}}}\right)\cdot {\text{V}}_{\text{shallow}}$$ (8)

$$\text{fused}=\text{concat}\left({\text{fused}}_{\text{shallow}\to \text{deep}},{\text{fused}}_{\text{deep}\to \text{shallow}}\right)$$ (9)

In Equations (7), (8), and (9), Q,Kand V denote the query, key, and value tensors of the feature maps, while shallow and deep represent the shallow and deep layers, respectively.

**Fig 6:**
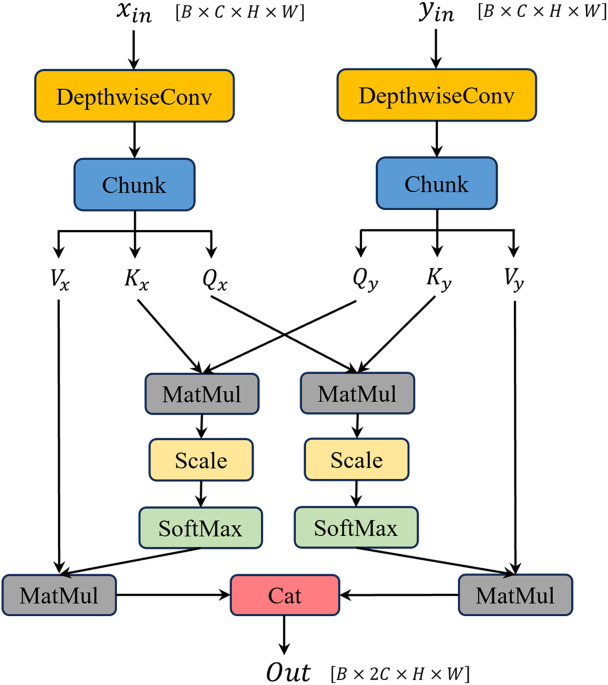
Bidirectional Depthwise Cross-Attention (BDCA) module.

The BDCA module is employed in the fusion part of the neck network in YOLOv11-WBD, adopting a weighted aggregation output to replace traditional concatenation operations. The detailed network architecture is illustrated in Fig 6.

The BDCA module implements feature map fusion using the concept of cross-attention, which requires feature maps to share identical dimensions. The “qkv” channel count defaults to matching the input feature map’s channel count. Subsequent ablation experiments indicate that this module can achieve relatively significant improvements in model accuracy compared to traditional concatenation operations.

## 4. Experiments and results

### 4.1. Dataset introduction and evaluation metrics

The proposed YOLOv11-WBD model was evaluated on two datasets: NEU-DET [22] and GC10-DET [23].

**Fig 7:**
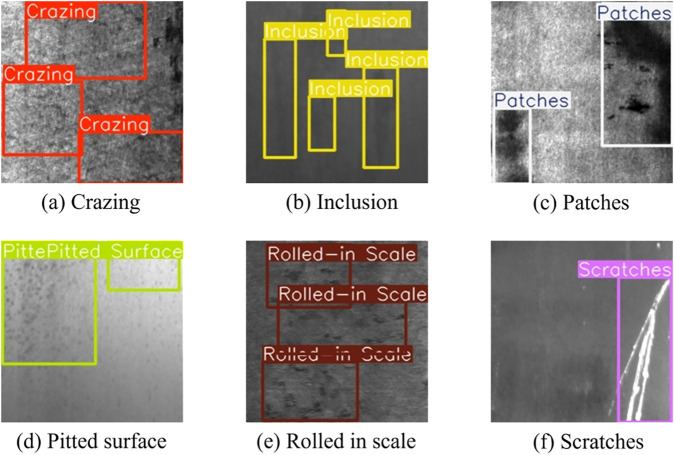
An example of the NEU-DET steel strip surface defect dataset.

The NEU-DET dataset is an open-source metal surface defect dataset published by Northeastern University, containing 1800 images of hot-rolled steel strip surfaces. As shown in Fig 7, it covers six types of defects: rolled-in scale (RS), patches (Pa), crazing (Cr), pitted surface (PS), inclusion (In), and scratches (Sc). It was randomly divided into a training set (1440 images), validation set (180 images), and test set (180 images) in an 8:1:1 ratio.

**Fig 8:**
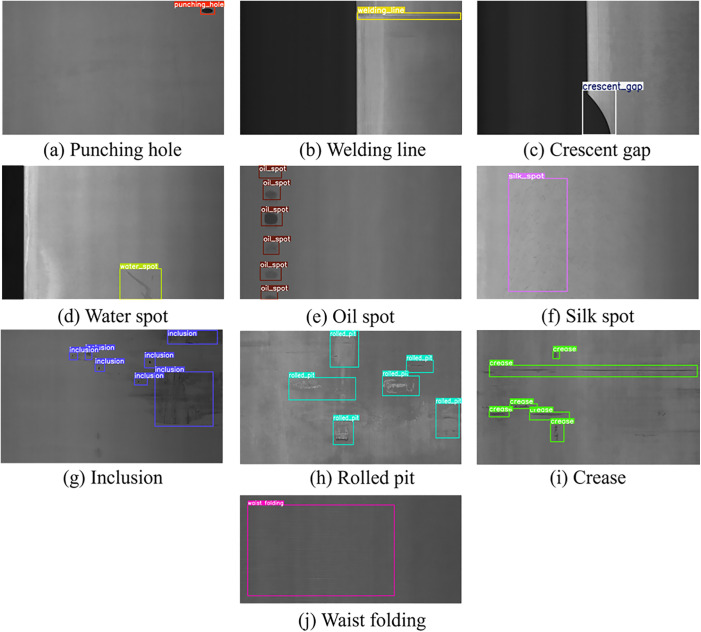
An example of the GC10-DET steel strip surface defect dataset.

The GC10-DET dataset is an open-source metal surface defect dataset released by the Institute of Automation, Chinese Academy of Sciences, containing 2294 images of steel plate surface defects from real industrial scenarios. As shown in Fig 8, it covers ten types of defects: punch (Pu), welding line (Wl), crescent gap (Cg), water spot (Ws), oil spot (Os), silk spot (Ss), inclusion (In), rolled pit (Rp), crease (Cr), and waist fold (Wf). It was also randomly divided into a training set (1836 images), validation set (229 images), and test set (229 images) in an 8:1:1 ratio.

To measure model accuracy, we adopted the mAP metric under the COCO evaluation standard [24]. Relevant calculation formulas are shown in Equations (10) to (13).


$$P=\frac{TP}{TP+FP}$$ (10)

$$R=\frac{TP}{TP+FN}$$ (11)

$$AP=\;\int_{0}^{1}P(R)dR$$ (12)

$$MAP=\;\frac{1}{n}\sum {\mathrm{\backslash nolimits}}_{i=1}^{n}{AP}_{i}$$ (13)

Where: TP (True Positive) denotes the number of positive samples correctly predicted; FP (False Positive) denotes the number of negative samples incorrectly predicted as positive; FN (False Negative) denotes the number of positive samples incorrectly predicted as negative. Average Precision (AP) is obtained by integrating the P-R curve, which is generated from precision and recall rates of detection results under different confidence levels. The final mAP value is the median of AP across all classes. The mAP calculated at an IoU threshold of 0.5 is denoted as mAP@0.5, a widely used metric in the defect detection field.

### 4.2. Experimental environment and parameter setting

The experimental hardware and software configurations are shown in Table 1. To ensure fairness and comparability of experimental results, no pre-trained weights were used. During the training phase, only Mosaic data augmentation was employed with a batch size set to 32. The Adam optimizer was adopted with 200 epochs, an initial learning rate of 0.001, complemented by a learning rate warm-up strategy to stabilize the training process. The learning rate was dynamically adjusted with an exponential decay rate of 0.97.

**Table 1:** Experimental basic environment configuration.

Parameters	Experimental Configuration
Operating system	Windows 11 ProPlus
GPU	NVIDIA GeForce RTX 4080 GPU
CPU	13th Generation Intel Core i7-13700KF
Display memory	16GB
Random access memory	48GB
Cuda environment	12.2
Deep learning frame work	PyTorch 2.0.1
Programming software	Python 3.10.11

### 4.3. Results and analysis

#### 4.3.1. Experiments results.

**Fig 9:**
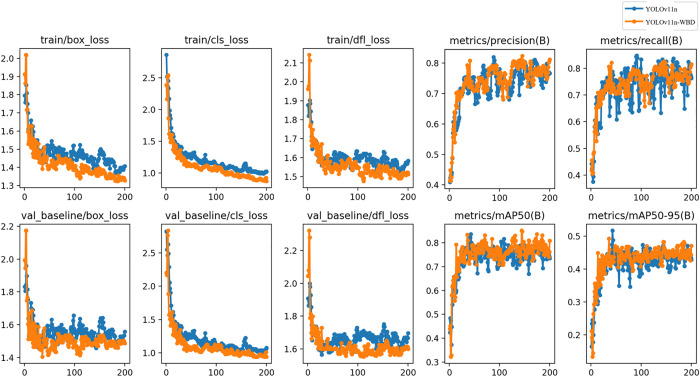
Training and validation losses and metric progression on NEU-DET dataset.

**Fig 10:**
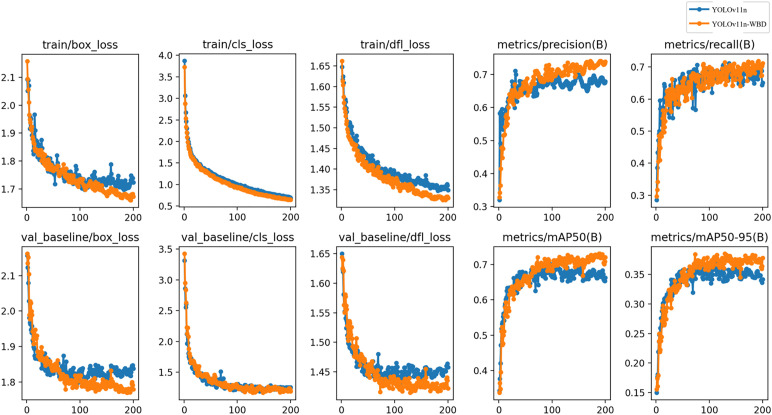
Training and validation losses and metric progression on GC10-DET dataset.

To validate the performance of YOLOv11-WBD in metal surface defect detection, we used YOLOv11n as the baseline model and YOLOv11n-WBD as the test model. The training processes on the NEU-DET dataset and GC10-DET dataset are shown in Figs 9 and 10, respectively.

In Figs 9 and 10, the box loss measures the deviation between predicted boxes and ground truth boxes, with lower values indicating more accurate detection localization; the classification loss evaluates the discrepancy between predicted classes and true classes, with lower values reflecting higher classification accuracy; the Distribution Focal Loss converts continuous coordinate predictions into discrete probability distributions for refined localization, with lower values representing superior coordinate prediction precision. Figs 9 and 10 show that after 200 training epochs, both YOLOv11n and YOLOv11n-WBD converge in the validation set, and YOLOv11n-WBD achieves higher mAP@0.5 than YOLOv11n in the validation set.

**Fig 11:**
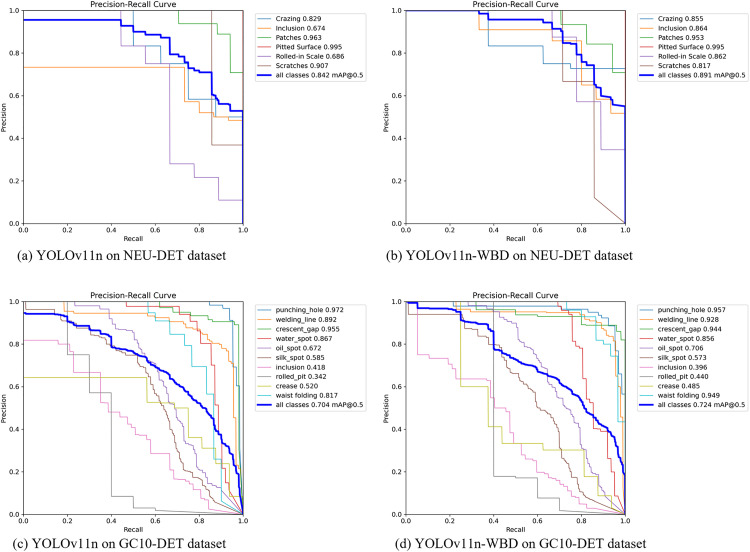
Precision-recall curve.

The average precision (AP) for various defect detections by YOLOv11n and YOLOv11n-WBD on the full NEU-DET dataset and full GC10-DET dataset is shown in Fig 11.

Fig 11 indicates that YOLOv11n-WBD outperforms YOLOv11n on both NEU-DET and GC10-DET datasets. On the NEU-DET dataset, YOLOv11n-WBD achieves a 5.8% improvement in mAP@0.5 over YOLOv11n; on the GC10-DET dataset, YOLOv11n-WBD achieves a 2.8% improvement in mAP@0.5 over YOLOv11n. Notably, substantial improvements in AP are observed for inclusions and rolled-in scale in NEU-DET, and for waist folds in GC10-DET. This demonstrates that the proposed WAMF, BEDU, and BDCA modules effectively enhance the baseline model’s representation capability and semantic mining effectiveness.

In summary, experimental results indicate that for metal surface defect detection scenarios, given sufficient training, YOLOv11n-WBD achieves superior detection accuracy compared to YOLOv11n. On the NEU-DET dataset, mAP@0.5 improves by 5.8%; on the GC10-DET dataset, mAP@0.5 improves by 2.8%.

#### 4.3.2. Comparative experiments.

**Fig 12:**
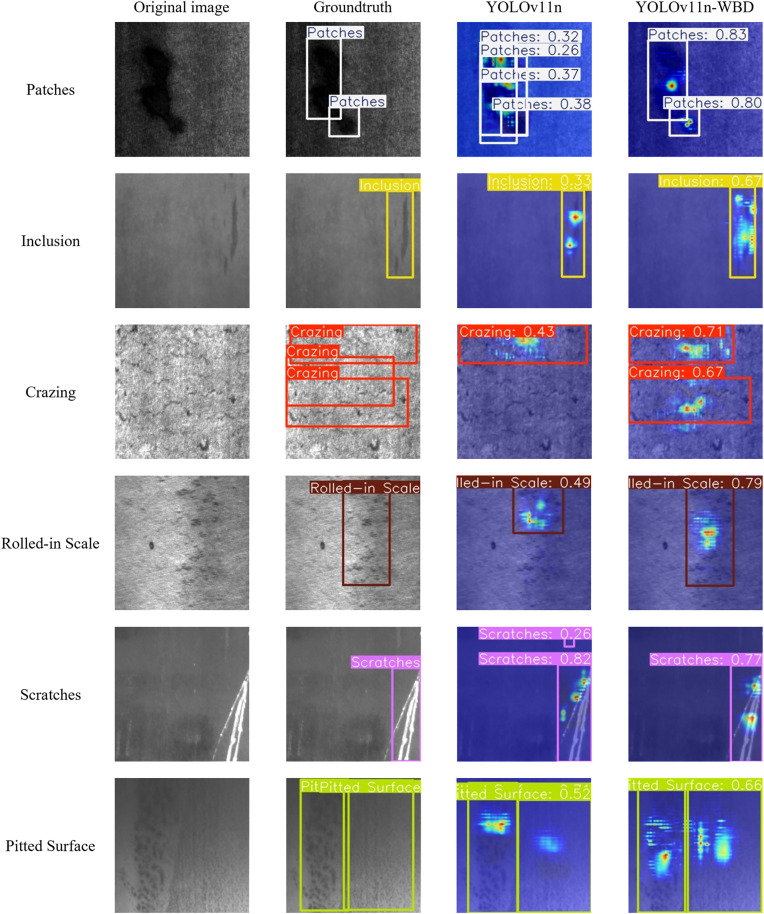
Heatmap comparison on NEU-DET datasets.

To intuitively evaluate the detection performance of YOLOv11n-WBD on metal surface defects, Fig 12 presents a comparison of detection results between YOLOv11n-WBD and YOLOv11n for six types of defect samples on the NEU-DET dataset. HiResCAM was used to generate heatmaps, visually illustrating the difference in attention focus on defect targets between the two YOLO models. The color intensity in the heatmaps is positively correlated with attention strength: brighter hues indicate higher saliency and a greater likelihood of containing the target.

Fig 12 shows that for the Patches, Inclusion, Scratches, and Pitted Surface categories in NEU-DET, both YOLOv11n and YOLOv11n-WBD achieve relatively accurate localization and classification. However, for Inclusion, Crazing, Rolled-in Scale, and Pitted Surface, YOLOv11n-WBD demonstrates higher confidence in detected defects compared to YOLOv11n. Moreover, the heatmaps of YOLOv11n-WBD align more closely with the target edges than those of YOLOv11n, indicating stronger detection stability.

**Fig 13:**
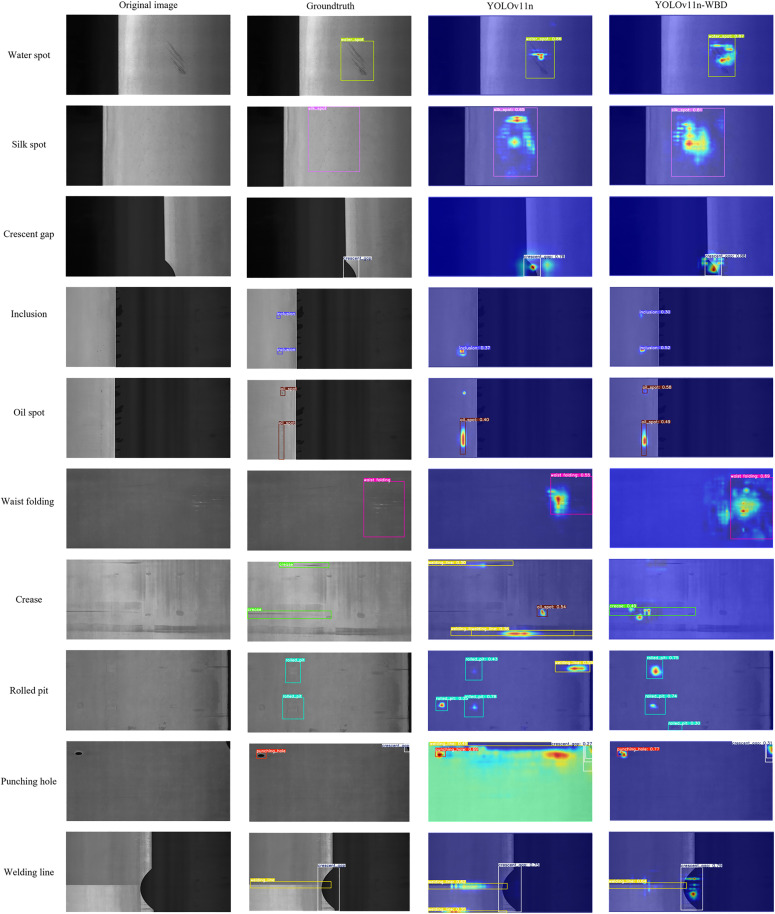
Heatmap comparison on GC10-DET dataset.

Fig 13 presents the comparison of detection results and heatmaps between YOLOv11n-WBD and YOLOv11n for ten types of defect samples on the GC10-DET dataset.

Fig 13 reveals that detecting some defects in the GC10-DET dataset is significantly more challenging than in NEU-DET. Examples include small-sized Inclusion targets, Waist folding with weak background contrast, and easily confusable categories like Welding line and Crease. For Water spot, Silk spot, Crescent gap, and Rolled pit, both YOLOv11n and YOLOv11n-WBD achieve relatively accurate localization and classification. However, for Inclusion and Oil spot defects appearing as small targets, YOLOv11n exhibited missed detections, while YOLOv11n-WBD correctly identified them. Furthermore, for easily confusable defects like Welding line and Crease, YOLOv11n is more prone to false positives compared to YOLOv11n-WBD. Additionally, for Crescent gap and Waist folding, the heatmaps of YOLOv11n-WBD align more closely with the core regions of the targets than those of YOLOv11n. Finally, for the Punching hole sample, the heatmap of YOLOv11n indicates significant interference affecting the model, whereas the heatmap of YOLOv11n-WBD shows the model was not severely affected.

To further verify the effectiveness and feasibility of the proposed model, it was compared with mainstream detection algorithms including YOLOv5 [25], YOLOv7 [26], and YOLOv8 [27] on both the NEU-DET and GC10-DET datasets. Experimental results are shown in Tables 2 and 3.

**Table 2:** Results of comparison experiments on dataset NEU-DET.

Model	Precision%	Recall%	mAP@50	Parameters/M	FLOPs/G
YOLOv5n [28]	66.8	70.8	72.3	1.8	4.2
YOLOv7_tiny	80.8	65.1	72.9	6.0	13.2
YOLOv8n	72.0	71.7	76.4	3.0	8.1
YOLOv11n	78.5	77.3	84.2	2.6	6.4
YOLOv11s	80.1	78.6	88.6	9.4	21.6
YOLOv11n-WBD	79.8	80.2	89.1	4.8	10.7

**Table 3:** Results of comparison experiments on dataset GC10-DET.

Model	Precision%	Recall%	mAP@50	Parameters/M	FLOPs/G
YOLOv5n	64.9	68.1	68.3	1.8	4.2
YOLOv7_tiny	59.0	63.8	62.6	6.0	13.2
YOLOv8n	61.9	66.5	68.3	3.0	8.1
YOLOv11n	68.8	67.3	70.4	2.6	6.4
YOLOv11s	70.4	67.9	72.5	9.4	21.6
YOLOv11n-WBD	70.1	68.2	72.4	4.8	10.7

The experimental results demonstrate that the YOLOv11n-WBD model performs excellently on both datasets. Compared to YOLOv5n, YOLOv7_tiny, YOLOv8n, and YOLOv11n, YOLOv11n-WBD exhibits superior accuracy. While compared to YOLOv11s, which has larger model capacity and higher algorithmic complexity, YOLOv11n-WBD achieves slightly higher accuracy on the NEU-DET dataset but slightly lower accuracy on the GC10-DET dataset.

In summary, the comparative experiments prove that for metal surface defect detection scenarios, YOLOv11n-WBD, benefiting from its effective feature representation and semantic mining capabilities, can more accurately capture the unique features of defects against complex backgrounds compared to other commonly used models in the YOLO series, achieving a better balance between accuracy and model complexity.

#### 4.3.3. Noise resistance capability analysis.

To evaluate the defect detection performance of YOLOv11n-WBD under varying noise intensity environments, this paper employs the Signal-to-Noise Ratio (SNR) metric [29] to quantify noise intensity. The calculation formula is shown in Equation (14).


$$SNR=\;10{log}_{10}\left(\frac{\sum_{i=1}^{H}\sum_{j=1}^{W}I_{signal}(i,j{)}^{2}}{\sum_{i=1}^{H}\sum_{j=1}^{W}I_{noise}(i,j{)}^{2}}\right)$$ (14)

In the equation, $I_{signal}(i,j)$ represents the pixel matrix of the original clear image, and $I_{noise}(i,j)$ represents the noise component matrix. A lower SNR value indicates stronger noise. Generally, low-intensity noise corresponds to SNR values greater than 30db, medium-intensity noise corresponds to the SNR range of 15 ~ 30db, high-intensity noise corresponds to SNR values less than 15db [30].

**Fig 14:**
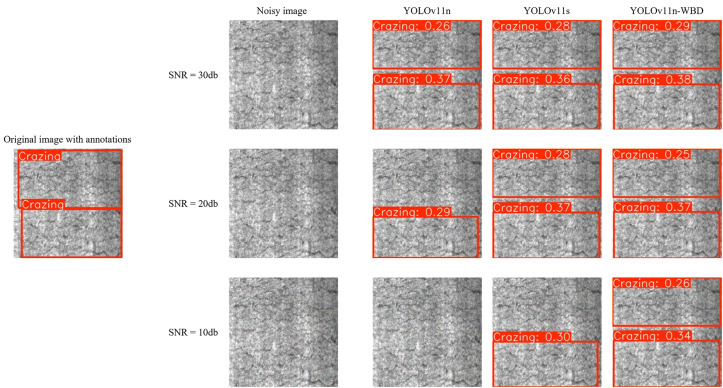
Comparative Analysis of Noisy Image Predictions on the NEU-DET dataset.

**Fig 15:**
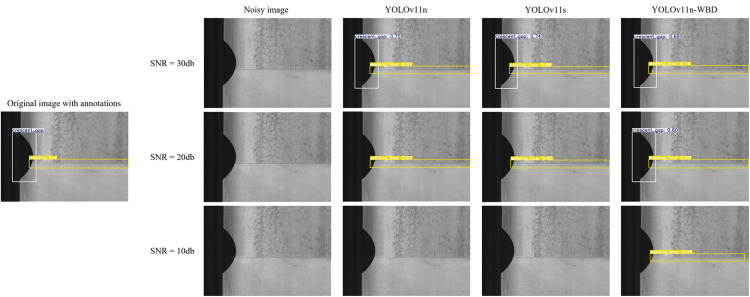
Comparative Analysis of Noisy Image Predictions on the GC10-DET dataset.

Actual operational conditions were simulated by adding Gaussian noise of different intensities to the original clear images. Figs 14 and 15 sequentially show the comparison of detection results by YOLOv11n, YOLOv11s, and YOLOv11n-WBD on the same sample after adding different noise intensities on the NEU-DET and GC10-DET datasets, respectively.

Figs 14 and 15 show that although noise of varying degrees was added to the metal surface inspection images, it did not significantly alter the inherent features of the original images. For example: for the sample in Fig 14, the characteristics of Crazing remain obvious before and after adding different noise intensities; for the sample in Fig 15, the characteristics of Crescent gap remain obvious. However, the three models exhibited different levels of noise tolerance. For instance: under low-intensity noise, all three models could correctly locate and identify Crazing, Crescent gap, and Welding line; under medium-intensity noise, YOLOv11n missed one Crazing defect in Fig 14 and one Crescent gap defect in Fig 15, while YOLOv11s, with its wider and deeper network architecture, also showed missed detections and decreased confidence in true positive targets. Nevertheless, YOLOv11n-WBD could still detect all defects normally; under high-intensity noise, YOLOv11n and YOLOv11s essentially lost their defect detection capability, and YOLOv11n-WBD also began to exhibit missed detections and decreased confidence in true positive targets. To further quantify noise tolerance, we compared YOLOv11n-WBD and the baseline model using the miss detection rate [31] as the metric, evaluating their performance under varying noise intensities on both the NEU-DET and GC10-DET datasets, as presented in Table 4.

**Table 4:** The comparison of model missed detection rates under different noise intensities.

Noise Intensity	Model	Missed detection rate on dataset NEU-DET	Missed detection rate on dataset GC10-DET
SNR = 30db	YOLOv11n	12%	18%
	YOLOv11n-WBD	8%	11%
SNR = 20db	YOLOv11n	54%	61%
	YOLOv11n-WBD	19%	24%
SNR = 10db	YOLOv11n	91%	97%
	YOLOv11n-WBD	68%	72%

In summary, the experimental results demonstrate that, compared to YOLOv11n and YOLOv11s, YOLOv11n-WBD, benefiting from its three improved core modules, exhibits stronger noise tolerance. It maintains a high defect detection capability even in moderate noise environments.

### 4.4. Ablation experiments

To verify the effectiveness of each module in the proposed improved algorithm, systematic ablation experiments were conducted on the NEU-DET and GC10-DET datasets, with results shown in Tables 5 and 6, respectively.

**Table 5:** Results of ablation experiments on dataset NEU-DET.

YOLOv11n	WAMF	BEDU	BDCA	mAP@50	Parameters/M	FLOPs/G
√				84.2	2.6	6.6
√	√			85.5	2.8	6.3
√		√		86.6	4.1	9.9
√			√	87.0	3.1	7.6
√	√	√		87.2	4.3	9.5
√	√		√	88.3	3.4	7.4
√		√	√	88.5	4.6	10.8
√	√	√	√	89.1	4.8	10.7

**Table 6:** Results of ablation experiments on dataset GC10-DET.

YOLOv11n	WAMF	BEDU	BDCA	mAP@50	Parameters/M	FLOPs/G
√				70.4	2.6	6.6
√	√			70.9	2.8	6.3
√		√		71.3	4.1	9.9
√			√	71.1	3.1	7.6
√	√	√		71.8	4.3	9.5
√	√		√	71.9	3.4	7.4
√		√	√	72.1	4.6	10.8
√	√	√	√	72.4	4.8	10.7

Tables 5 and 6 show that adding a single module to the baseline model improves detection accuracy on both datasets. Among them, the WAMF module, primarily by coupling traditional image enhancement with the shallow layers of the original YOLOv11 backbone network through wavelet decomposition, enhances model robustness while reducing complexity but contributes limitedly to accuracy improvement. In contrast, introducing the BDCA module achieves a relatively larger improvement in model accuracy at the cost of a slight increase in parameters and computational overhead by employing a bidirectional cross-attention mechanism. Additionally, introducing the BEDU module enhances the model’s ability to capture low-contrast and tiny targets. However, due to the limited proportion of such targets in the datasets, this results in a modest improvement in detection accuracy.

Introducing multiple modules to the baseline model further enhances detection accuracy on both datasets (accuracy surpassing the introduction of any single module), validating the collaborative enhancement effect of multiple modules. On the NEU-DET dataset, integrating all modules achieves optimal performance: mAP@50 is 89.1; precision is 79.8%; recall is 80.2%; model parameters are 4.8M; FLOPs are 10.7. On the GC10-DET dataset, integrating all modules achieves optimal performance: mAP@50 is 72.4; precision is 70.1%; recall is 68.2%; model parameters are 4.8M; FLOPs are 10.7.

In summary, the experimental results demonstrate that the three proposed modules enhance the model to varying degrees. When all three modules are simultaneously introduced into the original YOLOv11 model, they effectively improve detection accuracy while balancing model complexity and robustness.

## 5. Conclusion

This study addresses the challenge where mainstream object detection algorithms struggle to fully leverage their performance advantages under practical operational conditions due to constrained computational resources and image noise contamination. We propose the YOLOv11-WBD model for metal surface defect detection to balance detection accuracy, model complexity, and model robustness. Experimental results demonstrate:

1. The proposed WAMF module effectively achieves decoupling of low-frequency and high-frequency features and adaptive multi-band fusion, reducing interference from high-frequency noise and significantly enhancing model robustness.
2. The proposed BEDU module effectively fuses global and local information through dilated convolution with large receptive fields and a U-shaped skip connection structure, enhancing the model’s ability to capture low-contrast and tiny targets.
3. The proposed BDCA module maps deep and shallow features to a shared query/key/value embedding space via depthwise separable convolution. By employing a bidirectional cross-attention mechanism, it achieves a relatively larger improvement in model accuracy at the cost of a slight increase in parameters and computational overhead.

Comparative experiments and ablation studies were conducted on the NEU-DET and GC10-DET datasets. Evaluated using Precision, Recall, and mAP@0.5 metrics, the proposed model demonstrates superior generalization capability in metal surface defect detection scenarios compared to the baseline model. Furthermore, noise tolerance analysis experiments confirm that the model exhibits stronger noise tolerance, maintaining higher defect detection capability even in moderate noise environments.

## References

[1] Wu L, Hao HY, Song Y. A review of metal surface defect detection based on computer vision. Acta Automatica Sinica. 2024;50:1261–83. doi: 10.16383/j.aas.c230039

[2] Yu Y, Dong Y, Jiang Y, Wang F, Zhou Q, Ba P. Research on the Defect Detection Method of Steel-Reinforced Concrete Based on Piezoelectric Technology and Weight Analysis. Sensors (Basel). 2025;25(13):3844. doi: 10.3390/s25133844 40648102 PMC12251878

[3] Li J, Li M, Huang S, Wang G, Zhao X. Industrial Image Anomaly Detection via Synthetic-Anomaly Contrastive Distillation. Sensors (Basel). 2025;25(12):3721. doi: 10.3390/s25123721 40573607 PMC12197221

[4] Wang H, Xu X, Liu Y, Lu D, Liang B, Tang Y. Real-Time Defect Detection for Metal Components: A Fusion of Enhanced Canny–Devernay and YOLOv6 Algorithms. Applied Sciences. 2023;13(12):6898. doi: 10.3390/app13126898

[5] Wang X, Gao S, Guo J, Wang C, Xiong L, Zou Y. Deep Learning-Based Integrated Circuit Surface Defect Detection: Addressing Information Density Imbalance for Industrial Application. Int J Comput Intell Syst. 2024;17(1). doi: 10.1007/s44196-024-00423-w

[6] Li D, Yang P, Zou Y. Optimizing Insulator Defect Detection with Improved DETR Models. Mathematics. 2024;12(10):1507. doi: 10.3390/math12101507

[7] Li B, Gao Q. Defect Detection for Metal Shaft Surfaces Based on an Improved YOLOv5 Algorithm and Transfer Learning. Sensors (Basel). 2023;23(7):3761. doi: 10.3390/s23073761 37050821 PMC10098564

[8] Fişne A, Kalay A, Eken S. Fast and efficient computing for deep learning-based defect detection models in lightweight devices. J Intell Manuf. 2024. doi: 10.1007/s10845-024-02487-z

[9] Cheng Y, Liu D. AdIn-DETR: Adapting Detection Transformer for End-to-End Real-Time Power Line Insulator Defect Detection. IEEE Trans Instrum Meas. 2024;73:1–11. doi: 10.1109/tim.2024.3420265

[10] Zhang BF, Yu JH, Zhu XF, Sun ZF, Lu Y. Metal- POLO Detection Algorithm for Defects in Coaxial Packaged Metal Base. Laser Optoelectron Prog. 2024;61(22):2212003. doi: 10.3788/lop240829

[11] Zhu X, Liu J, Zhou X, Qian S, Yu J. Detection of irregular small defects on metal base surface of infrared laser diode based on deep learning. Multimed Tools Appl. 2023;83(7):19181–97. doi: 10.1007/s11042-023-16352-3

[12] Wang P, Wang W, Wang Y. Physically-based data augmentation for deep learning-enabled automated visual inspection of scratches. In: 2024 IEEE 20th International Conference on Automation Science and Engineering (CASE), 2024. 1644–9. doi: 10.1109/case59546.2024.10711456

[13] Wang H, Zha T, Nie L, Zhang J, Tang Y, Zhao Y. Improved Faster R-CNN-Based Contact Lens Surface Defect Detection. Laser & Optoelectronics Progress. 2023;60.

[14] Zheng J, Zhang T. Wafer Surface Defect Detection Based on Background Subtraction and Faster R-CNN. Micromachines (Basel). 2023;14(5):905. doi: 10.3390/mi14050905 37241529 PMC10223917

[15] Liu Y, He Y, Wu X, Wang W, Zhang L n, Lu H. Potato sprouting and surface damage detection method based on improved faster R - CNN. Transactions of the Chinese Society for Agricultural Machinery. 2024;55:371–8.

[16] Dong J, Guo Q, Chen L, Sang F. Review on Optimization Algorithms for One-Stage Metal Surface Defect Detection in Deep Learning. Computer Engineering and Application. 2025;61:72–89.

[17] Gao F, Zhu Q, Shao G, Su Y, Yang J, Yu X. A fast surface‐defect detection method based on Dense‐YOLO network. CAAI Trans on Intel Tech. 2025;10(2):415–33. doi: 10.1049/cit2.12407

[18] Liang T, Jiang S, Li Q, Ouyang B, Lu S. PCB surface defect dataset and detection based on YOLOv5s-P6SE. Computer Engineering and Science. 2025;47:276–87.

[19] Li Z, Kwan B-H, Tham M-L, Ng O-E, Wang PS-P. Abnormal Detection of Commutator Surface Defects Based on YOLOv8. Int J Patt Recogn Artif Intell. 2024;38(12). doi: 10.1142/s0218001424500137

[20] Cheng C, Cheng X, Li D, Zhang J. Drill pipe detection and counting based on improved YOLOv11 and Savitzky-Golay. Sci Rep. 2025;15(1):16779. doi: 10.1038/s41598-025-01776-8 40369135 PMC12078708

[21] Cheng S, Han Y, Wang Z, Liu S, Yang B, Li J. An Underwater Object Recognition System Based on Improved YOLOv11. Electronics. 2025;14(1):201. doi: 10.3390/electronics14010201

[22] Song K, Yan Y. A noise robust method based on completed local binary patterns for hot-rolled steel strip surface defects. Applied Surface Science. 2013;285:858–64. doi: 10.1016/j.apsusc.2013.09.002

[23] Lv X, Duan F, Jiang J-J, Fu X, Gan L. Deep Metallic Surface Defect Detection: The New Benchmark and Detection Network. Sensors (Basel). 2020;20(6):1562. doi: 10.3390/s20061562 32168887 PMC7146379

[24] Lin TY, Maire M, Belongie S, Hays J, Perona P, Ramanan D, et al. Microsoft COCO: Common Objects in Context. In: Proceedings of the Computer Vision – ECCV 2014, 2014. 740–55.

[25] Yu Q, Han Y, Han Y, Gao X, Zheng L. Enhancing YOLOv5 Performance for Small-Scale Corrosion Detection in Coastal Environments Using IoU-Based Loss Functions. JMSE. 2024;12(12):2295. doi: 10.3390/jmse12122295

[26] Wang CY, Bochkovskiy A, Liao HYM. YOLOv7: Trainable bag-of-freebies sets new state-of-the-art for real-time object detectors. undefined. 2022. doi: 10.48550/ARXIV.2207.02696

[27] Kumar P, Kumar V. Exploring the Frontier of Object Detection: A Deep Dive into YOLOv8 and the COCO Dataset. In: 2023 IEEE International Conference on Computer Vision and Machine Intelligence (CVMI), 2023. 1–6. doi: 10.1109/cvmi59935.2023.10464837

[28] Jaiswal SK, Agrawal R. A Comprehensive Review of YOLOv5: Advances in Real-Time Object Detection. IJIRCST. 2024;12(3):75–80. doi: 10.55524/ijircst.2024.12.3.12

[29] Wang Q, Luo H, Li Z, Ding Y, Xiong W. Analysis of signal-to-noise ratio of spatial heterodyne spectroscopy. Measurement. 2024;237:115180. doi: 10.1016/j.measurement.2024.115180

[30] Wang Y, Wu J, Yu Z, Hu J, Zhou Q. A structurally re-parameterized convolution neural network-based method for gearbox fault diagnosis in edge computing scenarios. Engineering Applications of Artificial Intelligence. 2023;126:107091. doi: 10.1016/j.engappai.2023.107091

[31] Tao H, Zheng Y, Wang Y, Qiu J, Stojanovic V. Enhanced feature extraction YOLO industrial small object detection algorithm based on receptive-field attention and multi-scale features. Meas Sci Technol. 2024;35(10):105023. doi: 10.1088/1361-6501/ad633d

